# Impact of corticosteroids on lung antibiotic resistance genes in patients with lower respiratory tract infections

**DOI:** 10.1128/spectrum.02080-25

**Published:** 2026-07-15

**Authors:** Jiawei Shen, Yan Hu, Xiaoyun Zou, Xiujuan Zhao, Shu Li, Yanwen Jiang, Fengxue Zhu

**Affiliations:** 1 Department of Critical Care Medicine, Peking University People’s Hospital, Beijing, People’s Republic of China; 2 Department of Respiratory and Critical Care Medicine, Peking University International Hospital 594822, Beijing, China; 3 Department of Critical Care Medicine, Peking University People's Hospital, Women and Children's Hospital, Qingdao University 12593, Qingdao, Shandong, China; Guizhou Medical University, Guiyang, China; New York Presbyterian/Weill Cornell Medical Center, New York, New York, USA

**Keywords:** corticosteroids, drug resistance, respiratory tract infections, metagenomics

## Abstract

Lower respiratory tract infections (LRTIs) are a major global health concern, complicated by rising antibiotic resistance driven by antibiotic resistance genes (ARGs). Despite its role in the treatment of respiratory diseases, the impact of corticosteroids on ARGs in LRTI patients remains underexplored. Bronchial alveolar lavage (BAL) samples were collected from LRTI patients from two intensive care units (ICUs). Patients were classified into the corticosteroid group (CS group) and the non-corticosteroid group (NCS group) based on corticosteroid use. Next-generation sequencing assessed ARGs and associated microbes, with multivariable logistic regression analyzing the relationship between corticosteroid therapy and ARG accumulation. Ninety-one patients were recruited; the CS group (*n* = 57) exhibited a distinct ARG profile, marked by higher alpha-diversity and increased prevalence of ARGs than the NCS group (*n* = 34). The duration of corticosteroid therapy was positively associated with ARG accumulation, with individuals receiving treatment for more than 30 days exhibiting the highest ARG burden. The duration of corticosteroid therapy and the underlying hematological diseases were two independent risk factors for ARG accumulation. Our data provide new evidence that, in patients with LRTIs, extended corticosteroid use is associated with the accumulation of ARGs and modifications in the microbial composition of the lower respiratory tract.

**IMPORTANCE:**

This research provides new evidence that prolonged use of corticosteroid drastically increases antibiotic resistance genes (ARGs) in the lungs of LRTI patients. It reveals a duration-dependent accumulation of ARGs, notably for common broad-spectrum antibiotics. These findings highlight the need to consider ARG burden when evaluating corticosteroid prescribing practices in patients with lower respiratory tract infections.

## INTRODUCTION

Lower respiratory tract infections (LRTIs) represent a significant global health burden, associated with considerable morbidity, mortality, and healthcare costs (1, 2). Antibiotics were essential therapies for the treatment of LRTIs. However, the escalating prevalence of antibiotic resistance has complicated management strategies and undermined clinical outcomes (3). Antibiotic resistance genes (ARGs) serve as key genetic elements driving resistance phenotypes, often propagating through microbial communities in response to antibiotic exposure and selective environmental pressures (4).

Corticosteroids are extensively employed in clinical practice due to their potent anti-inflammatory and immunosuppressive effects, particularly in managing chronic pulmonary conditions (5, 6) (including asthma, chronic obstructive pulmonary disease), non-pulmonary diseases (7, 8) (autoimmune diseases and graft versus host diseases after solid organ transplantation and hematopoietic stem cell transplantations), acute respiratory distress syndromes (9), and COVID-19 pneumonia (10). Despite their clinical efficacy in reducing airway inflammation and facilitating symptomatic relief, recent evidence suggests alterations in pulmonary microbiota composition following corticosteroid administration. Corticosteroids affect multiple aspects of host immunity, including reduced neutrophil function, impaired mucosal barrier integrity, and decreased antimicrobial peptide production (11). These effects create conditions that may promote ARG accumulation through various mechanisms. First, immunosuppression allows prolonged colonization of bacteria, thus providing opportunity for horizontal gene transfer (12). Moreover, patients receiving corticosteroids often need prolonged antibiotic therapy, which will create selective pressure favoring ARG-carrying organisms (13). While previous studies have documented corticosteroid-associated microbial dysbiosis (14, 15) and accumulation of multidrug-resistant bacteria in immunocompromised patients (16), no research has directly quantified ARG burden in corticosteroid-treated populations.

Understanding the nuanced role of corticosteroids in ARG dynamics is vital for optimizing therapeutic strategies and informing antimicrobial stewardship in pulmonary infections. In this paper, by using prospectively collected bronchial alveolar lavage (BAL) samples from LRTI patients with or without previous therapy of corticosteroids, we aim to explore the effects on ARGs in BAL samples of corticosteroids with metagenomic sequencing analysis.

## MATERIALS AND METHODS

### Patient recruitment

**Fig 1:**
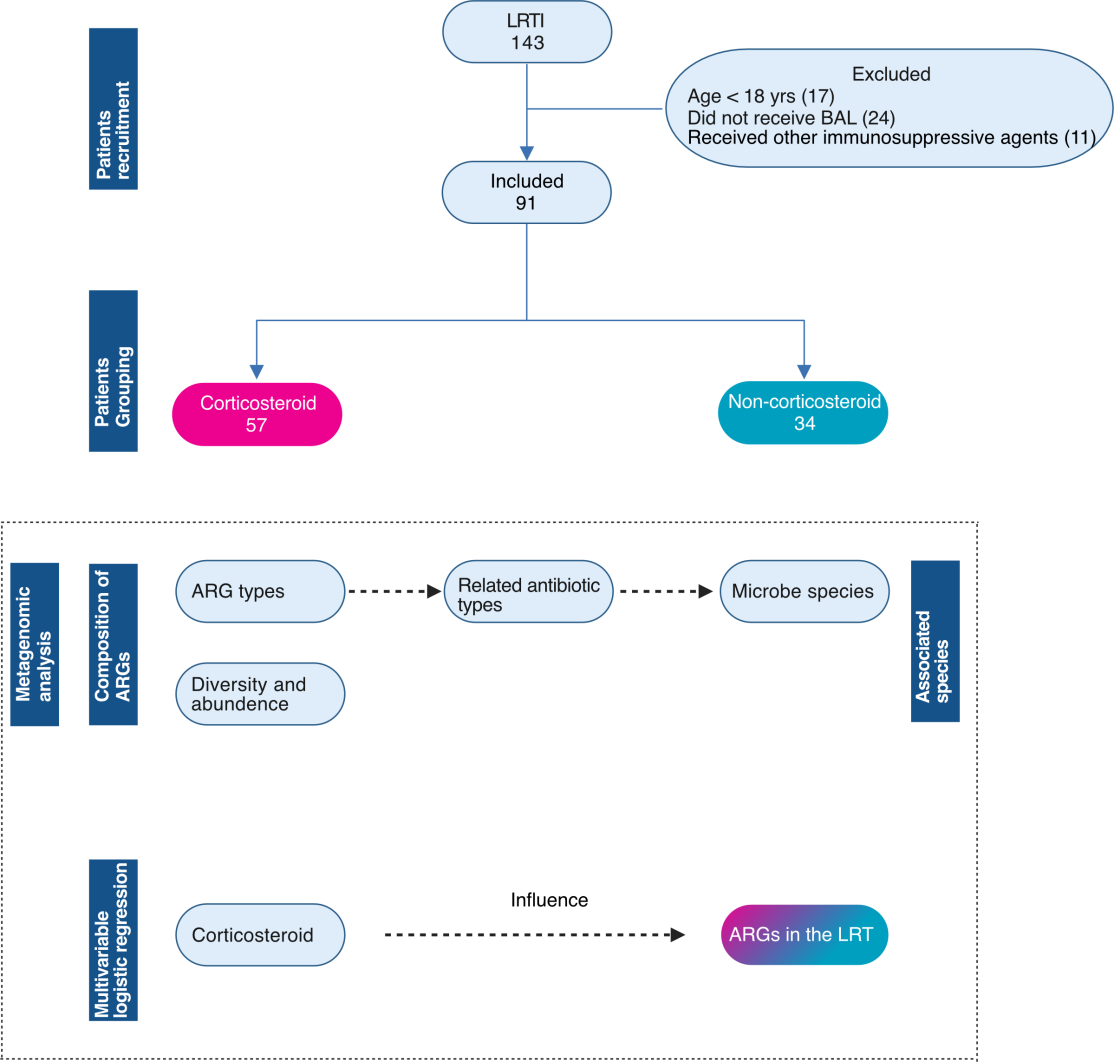
Flow chart of study design and patient recruitment. ARG, antibiotic resistance gene; LRTI, lower respiratory tract infection; CS, corticosteroids; NCS, non-corticosteroids.

Our study examined male and female patients, and similar findings are reported for both sexes. From January 2021 to December 2024, we prospectively collected patients diagnosed with LRTI from all the patients admitted to ICU in the two participating hospitals (Peking University People’s Hospital and Peking University International Hospital). The criteria for LRTI were as follows (study flowchart in Fig. 1):

1. Clinical criteria: (i) new onset of purulent sputum, change in character of sputum, increased respiratory secretions, or increased suctioning requirements; (ii) dyspnea, tachypnea, or new onset or worsening cough; (iii) rales or bronchial breath sounds; (iv) worsening gas exchange (PaO_2_/FiO_2_ ≤ 300); and (v) increased oxygen requirements or increased ventilator demand.
2. Radiology criteria: new infiltrates, consolidation, cavitation, ground-glass opacity, or interstitial changes.

Patients who met any one of the clinical criteria and imaging criteria were considered to have LRTI.

The exclusion criteria were as follows: (i) age <18 years old; (ii) received immunosuppressive agents other than corticosteroids; and (iii) declined to participate in the study or declined BAL.

Patients were grouped by whether they received corticosteroids (including inhaled, intravenous, or oral dosage forms) within 1 month before ICU admission, named corticosteroid group (CS group, for those who received corticosteroids) or non-corticosteroid group (NCS group, for those who did not receive corticosteroids).

### Baseline characteristics and lab results

For patients that participated in this study, we collected their baseline data including demographic variables (including age, gender, sequential organ failure assessment [SOFA] score, intubation rates, comorbidities, inflammatory cytokines, infection biomarkers, blood cell counts, and prior history of hospitalization, LRTI, and antibiotic use). All the samples were collected within 24 h of ICU admission. For patients receiving different steroid formulations, we converted all doses to methylprednisolone equivalents using standard conversion ratios: prednisone (×1.25), dexamethasone (×0.15), hydrocortisone (×5), and inhaled budesonide (×40 for systemic equivalent). Daily dosage was calculated as the average methylprednisolone-equivalent dose over the treatment period.

### BAL sample collection and metagenomic next-generation sequencing processing

All patients in this study received BAL within 24 h of ICU admission using a standardized protocol at both centers. For intubated patients, the bronchoscope was introduced through the endotracheal tube and wedged in the affected subsegmental bronchus. For non-intubated patients, bronchoscopy was performed under conscious sedation with topical lidocaine via the nasal route. Three 20 mL aliquots of sterile saline (total 60 mL) were instilled and retrieved by gentle suction. The first aliquot was discarded to reduce upper airway contamination. At least 5 mL of pooled BALF from the remaining aliquots was used for mNGS analysis. BALF samples were examined using RNA-based metagenomic next-generation sequencing (mNGS) to facilitate the identification of microorganisms. RNA was isolated from BALF samples, and cDNA libraries were created via reverse transcription and sequenced using the NextSeq 1000 System (150 bp paired-end reads; Illumina) to generate sequencing data for subsequent analysis. Sequencing data were processed using “Kneaddata” (v0.11.0) to eliminate host contaminants. Identification of ARGs was performed with RGI (the Resistance Gene Identifier, v6.0.3) using the BWT method with reference to CARD (the Comprehensive Antibiotic Resistance Database, v4.0.0). Identified sequences were then examined using the “Kraken2” (v2.1.2) to determine the taxonomy of species present in the BALF sample that contained the specified ARGs.

### Mobile genetic element analysis

To explore horizontal gene transfer potential, we analyzed mobile genetic elements (MGEs) directly from quality-filtered, host-depleted reads. Assembly-based approaches were avoided. BAL samples carry low microbial biomass, and the resulting fragmented contigs are unreliable for MGE annotation. Cleaned reads were mapped against mobileOG-db (v1.00), a curated database covering five MGE categories: plasmids, transposons, integrons, insertion sequences, and phages. Alignment mapping was performed using BWA-MEM (v0.7.17) with a minimum seed length of 19 (-k 19) and an alignment score threshold of 30 (-T 30). Reads with mapping quality below 20 were excluded.

ARG-MGE co-occurrence was determined by paired-read proximity analysis. Read pairs in which one mate mapped to an ARG (RGI v6.0.3, CARD v4.0.0) and the other mapped to an MGE gene in mobileOG-db were classified as MGE-associated. This exploits the 300–500 bp insert size of paired-end reads to infer genomic co-localization without assembly. ARGs with no MGE-associated mate reads were classified as chromosomal.

### Statistical analysis

All statistical analyses were performed in Python (v 3.9.6) using SciPy (v 1.15.1) and visualized with Matplotlib (v3.1.0) and Seaborn (v 0.13). Continuous variables were presented as medians (interquartile ranges), and categorical variables were presented as numbers (percentages). A comparison of categorical variables was achieved using the χ2 test. Comparison of two groups of continuous variables was performed using the Kruskal-Wallis H test. All tests were two-sided, and *P* < 0.05 was considered statistically significant. Alpha-diversity and beta-diversity of ARGs were calculated and displayed via non-metric multidimensional scaling (NMDS) using scikit-bio (v 0.6.3).

Sample size was determined by feasibility within the prospective collection period rather than by a formal *a priori* power calculation. Post-hoc power analysis using the observed effect size (Cohen’s d = 0.54) for ARG count differences between CS and NCS groups indicated 70% statistical power at α = 0.05 for detecting medium-sized effects with the achieved sample sizes (*n* = 57 in the CS group, *n* = 34 in the NCS group).

## RESULTS

### Patients

One hundred forty-three patients with an LRTI diagnosis were admitted to the two study centers during the study period. After exclusion for non-adult patients (*n* = 17), those who declined BAL (*n* = 24), or those who received immunosuppressive agents other than corticosteroids before admission (*n* = 11), 91 patients were recruited as participants. According to their baseline application of corticosteroids, patients were divided into the CS group (*n* = 57) and the NCS group (*n* = 34) (Fig. 1).

Patients in the two groups had similar demographical characteristics (Table 1; e.g., age or gender distribution) and similar disease severity (SOFA score, CS vs NCS: 5 [3–9] vs 4 [3–7], *P* = 0.396). Patients in the CS group had lower levels of C-reactive protein (CS vs NCS: 39.20 [10.35–58.63] mg/L vs 89.50 [45.80–142.20] mg/L, *P* = 0.025), serum IL-6 (CS vs NCS: 9.72 [3.32–14.80] pg/mL vs 43.28 [20.94–130.45] pg/mL, *P* = 0.002), TNF-α (CS vs NCS: 0 [0–2.26] pg/mL vs 15.34 [7.35–20.56] pg/mL, *P* = 0.004), and lymphocyte (CS vs NCS: 0.28 [10.35–58.63] × 10^9^/L vs 89.50 [45.80–142.20] × 10^9^/L, *P* = 0.025) than those in the NCS group. White blood cell counts (CS vs NCS: 2.5 [10.20–13.60] × 10^9^/L vs 4.92 [2.85–7.14] × 10^9^/L, *P* = 0.031) and granulocyte counts (CS vs NCS: 10.2 [6.15–12.10] × 10^9^/L vs 3.85 [2.05–5.99] × 10^9^/L, *P* < 0.001) were higher in the CS group.

**TABLE 1:** Characteristics of patients^*a*^^,^^*b*^

	Corticosteroid (CS)	Non-corticosteroid (NCS)	*P*
	(*n* = 57)	(*n* = 34)	
Age (y)	50 (22–58)	56 (38–61)	0.177
Female (%)	27 (47.37)	12 (35.29)	0.26
SOFA score	5 (3–9)	4 (3–7)	0.369
Underlying disease (%)			0.082
Autoimmune disease	13 (22.81)	7 (20.59)	
Asthma	5 (8.77)	2 (5.88)	
Bronchiectasis	5 (8.77)	9 (26.47)	
COPD	22 (38.60)	6 (17.65)	
Hematological disease	12 (21.05)	10 (29.41)	
Intubation	29 (50.88)	14 (41.18)	0.085
Max temperature, ℃	37.2 (36.2–37.6)	37.8 (37.2–38.6)	0.544
C-reactive protein, mg/L	39.20 (10.35–58.63)	89.50 (45.80–142.20)	0.025
Procalcitonin, ng/mL	2.32 (0.12–7.32)	1.58 (1.17–5.23)	0.396
Serum cytokines			
IL-2, pg/mL	7.14 (2.94–11.63)	32.42 (20.79–80.00)	0.176
IL-4, pg/mL	6.03 (1.57–13.51)	27.43 (15.78–90.62)	0.438
IL-6, pg/mL	9.72 (3.32–14.80)	43.28 (20.94–130.45)	0.002
IL-10, pg/mL	8.11 (2.45–12.74)	28.89 (19.13–113.16)	0.046
IFN-γ, pg/mL	5.36 (2.92–12.56)	31.12 (17.02–97.25)	0.381
TNF-α, pg/mL	0 (0–2.26)	15.34 (7.35–20.56)	0.004
Blood cell counts			
WBC, ×10^9^/L	12.50 (10.20–13.60)	4.92 (2.85–7.14)	0.031
Lymphocyte, ×10^9^/L	0.28 (0.15–0.55)	0.72 (0.61–0.85)	<0.001
Granulocyte, ×10^9^/L	10.20 (6.15–12.10)	3.85 (2.05–5.99)	<0.001
Hemoglobin, g/dL	80.50 (70.00–93.00)	76.80 (65.00–90.00)	0.433
Platelets, ×10^9^/L	43.00 (25.00–61.00)	38.00 (18.00–51.00)	0.539
Hospitalization within prior year (%)	38 (66.67)	19 (55.88)	0.301
Prior LRTI episodes within year (%)	32 (56.14)	16 (47.06)	0.394
Prior antibiotic use within 30 days (%)	45 (78.95)	25 (73.53)	0.55
Duration of antibiotic use	7.50 (3.00–10.00)	4.00 (2.50–5.50)	<0.001
Classes of antibiotics (%)			
Aminoglycoside	3 (5.26)	2 (5.9)	0.9
Beta-lactam	12 (21.1)	3 (8.8)	0.155
Carbapenems	6 (10.5)	2 (5.9)	0.705
Fluoroquinolone	17 (29.8)	11 (32.4)	0.818
MLSB	4 (7.0)	6 (17.6)	0.166
Tetracycline	3 (5.3)	0 (0.0)	0.29
Others	2 (3.5)	0 (0.0)	0.527
Corticosteroid route (%)			
Intravenous	38 (66.67)	N.A.	
Oral	11 (19.29)	N.A.	
Inhaled	8 (14.04)	N.A.	

^*a*^ Values are given as median (interquartile range) or mean ± standardized deviation or number (%).

^*b*^ FiO_2_, fraction of inspired oxygen; GvHD, graft versus host disease; HLA, human leukocyte antigen; HSCT, hematopoietic stem cell transplantation; IL, interleukin; IFN-γ, interferon-γ; LRTI, lower respiratory tract infection; PaO_2_, partial pressure of oxygen; MLSB, macrolide–lincosamide–streptogramin; N.A., not applicable; SOFA, sequential organ failure assessment; TNF, tumor necrosis factor; WBC, white blood cell.

The percent of antibiotic therapy within 30 days before study was not significantly different between the two groups (CS vs NCS: 45 [78.95%] vs 25 [73.53], *P* = 0.550). However, patients in the CS group had a longer duration of antibiotic use (CS vs NCS: 7.50 [3.00–10.00] vs 4.00 [2.50–5.50], *P* < 0.001). Corticosteroid use before admission was driven by underlying conditions: COPD (38.6%), autoimmune diseases (22.8%), and hematological diseases, including post-transplant complications (21.1%).

### ARG characteristics of CS and NCS groups

**Fig 2:**
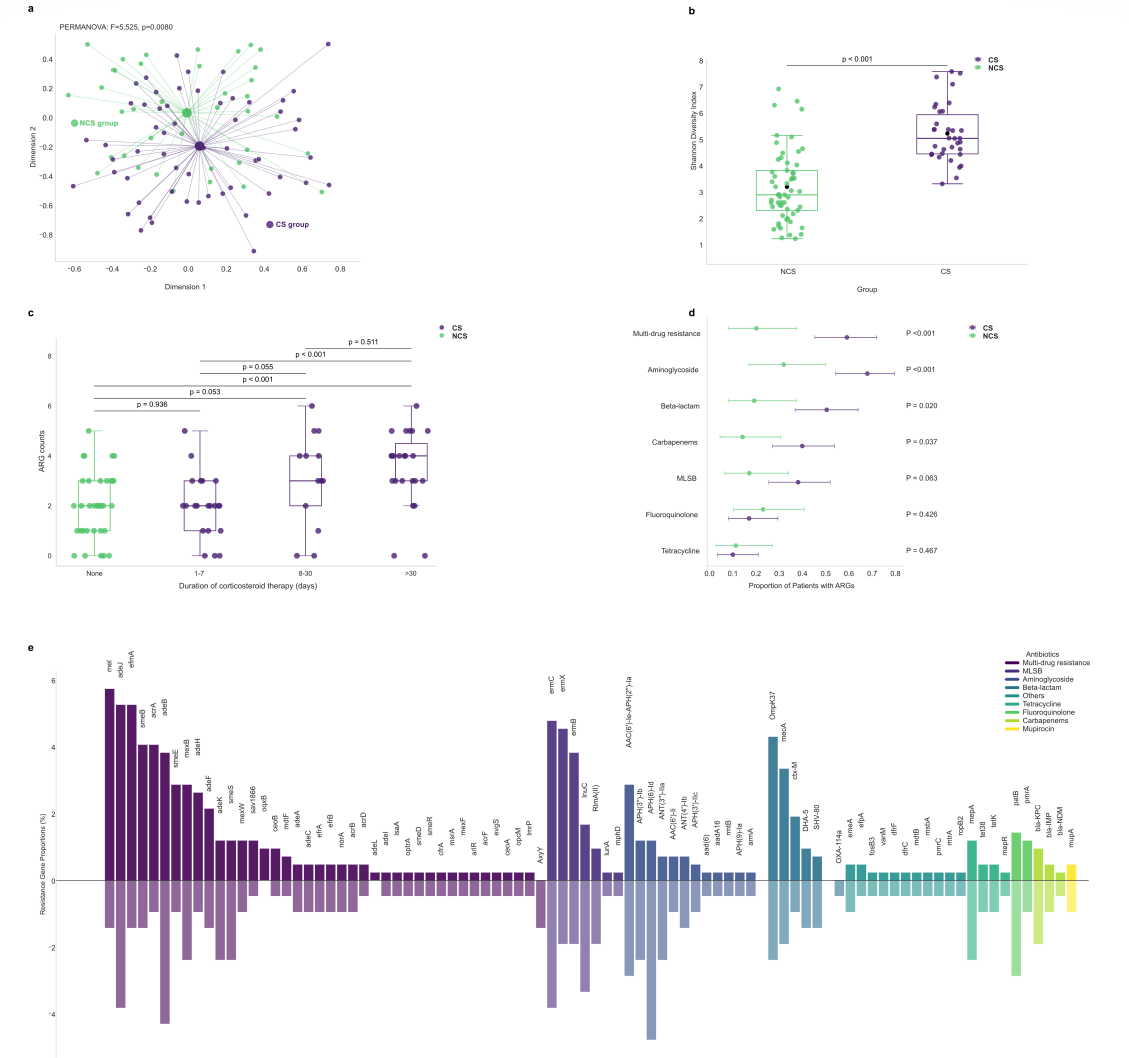
ARG characteristics of CS and NCS groups. (**a**) Composition of ARGs between two groups, evaluated with PERMANOVA test to describe the beta-diversity of ARGs. PERMANOVA F = 5.525, *P* = 0.008. (**b**) Comparison of ARG diversity between two groups, evaluated with Shannon diversity index to describe the alpha-diversity. *P* < 0.001. (**c**) ARG counts between the NCS group and patients with different durations of corticosteroid therapy. Significant differences: >30 days of CS vs NCS: 81 vs 69, *P* < 0.001, >30 days of CS vs 1–7 days of CS: 81 vs 42, *P* < 0.001. (**d**) Proportion of patients with types of ARGs. Patients in the CS group had significantly more ARGs that contributed to multidrug resistance (CS vs NCS: 59.65% vs 20.59%, *P* < 0.001), aminoglycoside resistance (CS vs NCS: 68.42% vs 32.35%, *P* < 0.001), beta-lactam resistance (CS vs NCS: 50.88% vs 20.56%, *P* = 0.020), and carbapenem resistance (CS vs NCS: 40.35% vs 14.71%, *P* = 0.037). (**e**) Distributions of all identified ARGs in the two groups. ARGs, antibiotic resistance genes; CS, corticosteroid; MLSB, macrolide–lincosamide–streptogramin B; NCS, non-corticosteroid.

Comparison of the composition of ARGs between two groups was achieved using PERMANOVA. The result showed that, compared to the NCS group, patients in the CS group had a different composition of ARGs in the lower respiratory tract (evaluated using beta-diversity, PERMANOVA F = 5.525, *P* = 0.008, Fig. 2a) and higher alpha-diversity (evaluated using Shannon diversity index, *P* < 0.001, Fig. 2b). Patients with longer duration of corticosteroid therapy had higher ARG counts than those who did not receive corticosteroids (Fig. 2c). The difference was significant only in patients who received corticosteroid therapy for more than 30 days and in patients without corticosteroid therapy (>30 days of CS vs NCS: 81 vs 69, *P* < 0.001) and in those with 1–7 days of corticosteroid therapy (>30 days of CS vs 1–7 days of CS: 81 vs 42, *P* < 0.001). In detail (Fig. 2d), patients in the CS group had significantly more ARGs associated with multidrug resistance (CS vs NCS: 59.65% vs 20.59%, *P* < 0.001), aminoglycoside resistance (CS vs NCS: 68.42% vs 32.35%, *P* < 0.001), beta-lactam resistance (CS vs NCS: 50.88% vs 20.56%, *P* = 0.020), and carbapenem resistance (CS vs NCS: 40.35% vs 14.71%, *P* = 0.037). There were a total of 86 ARGs, classified into nine classes of antibiotic resistance, identified in the samples. Their distribution in the CS and NCS groups is depicted in Fig. 2e.

### ARG-related microbe compositions in the CS and NCS groups

**Fig 3:**
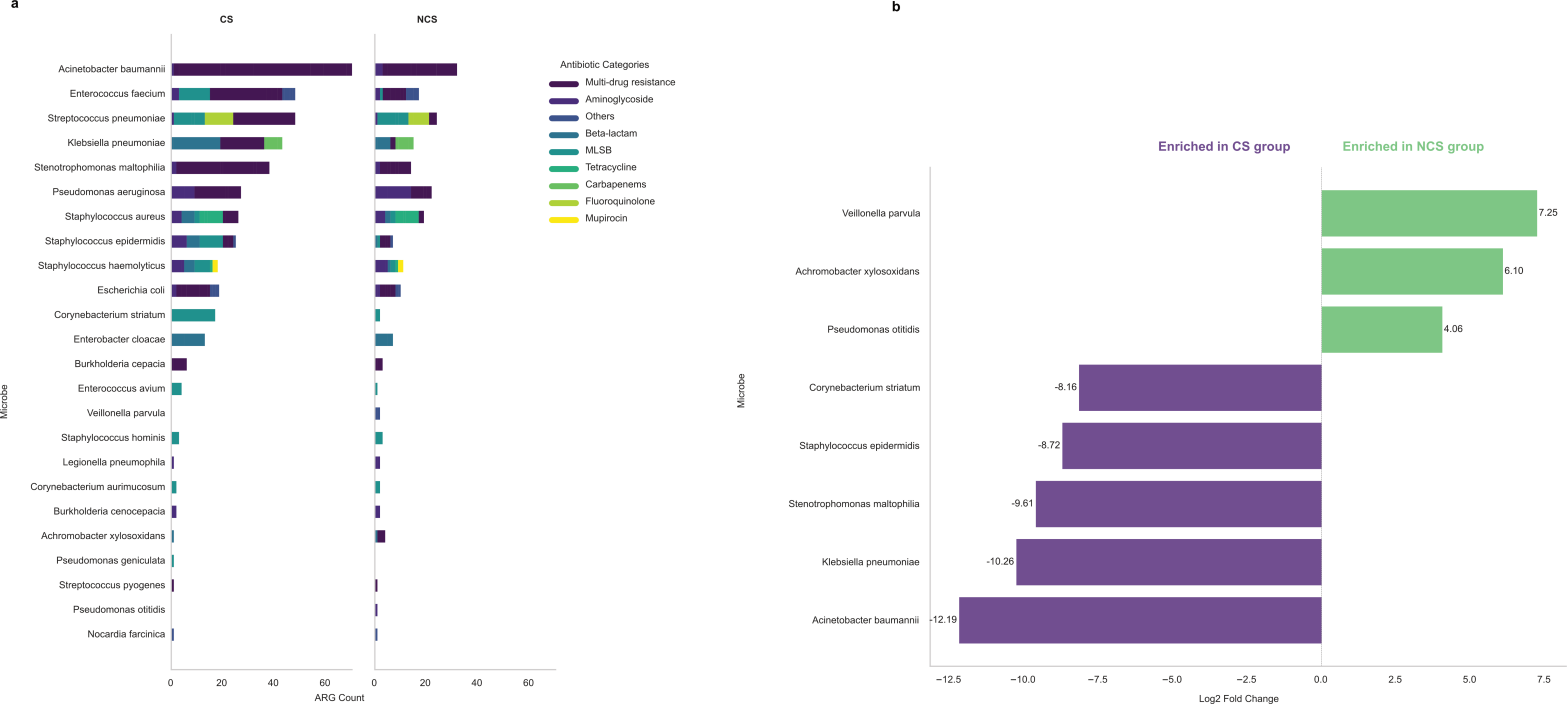
Microbes carrying the identified ARGs. (**a**) Compositions of ARG-carrying microbes in the two groups, ordered by the number of ARGs carried by each microbe in the CS group. (**b**) Differential analysis of ARG-carrying microbes between the two groups. Threshold for significance: log2 fold change >2, *P* < 0.05. ARGs, antibiotic resistance genes; CS, corticosteroid; NCS, non-corticosteroid.

We then described the compositions of microbes in the two groups that carried the ARGs (Fig. 3a). Microbes with various ARGs were present in both the CS and NCS groups, and the microbes of patients in the CS group had a greater overall number of ARG classes than those in the NCS group. In differential analysis, ARG-associated microbes that were enriched (threshold for significance: log2 fold change >2, *P* < 0.05) in the two groups are shown in Fig. 3b. *Acinetobacter baumannii*, *Klebsiella pneumoniae*, *Stenotrophomonas maltophilia*, *Staphylococcus epidermidis*, and *Corynebacterium striatum* were enriched in the CS group, while *Veillonella parvula*, *Achromobacter xylosoxidans*, and *Pseudomonas otitidis* were enriched in the NCS group.

### Mobile genetic element analysis

**Fig 4:**
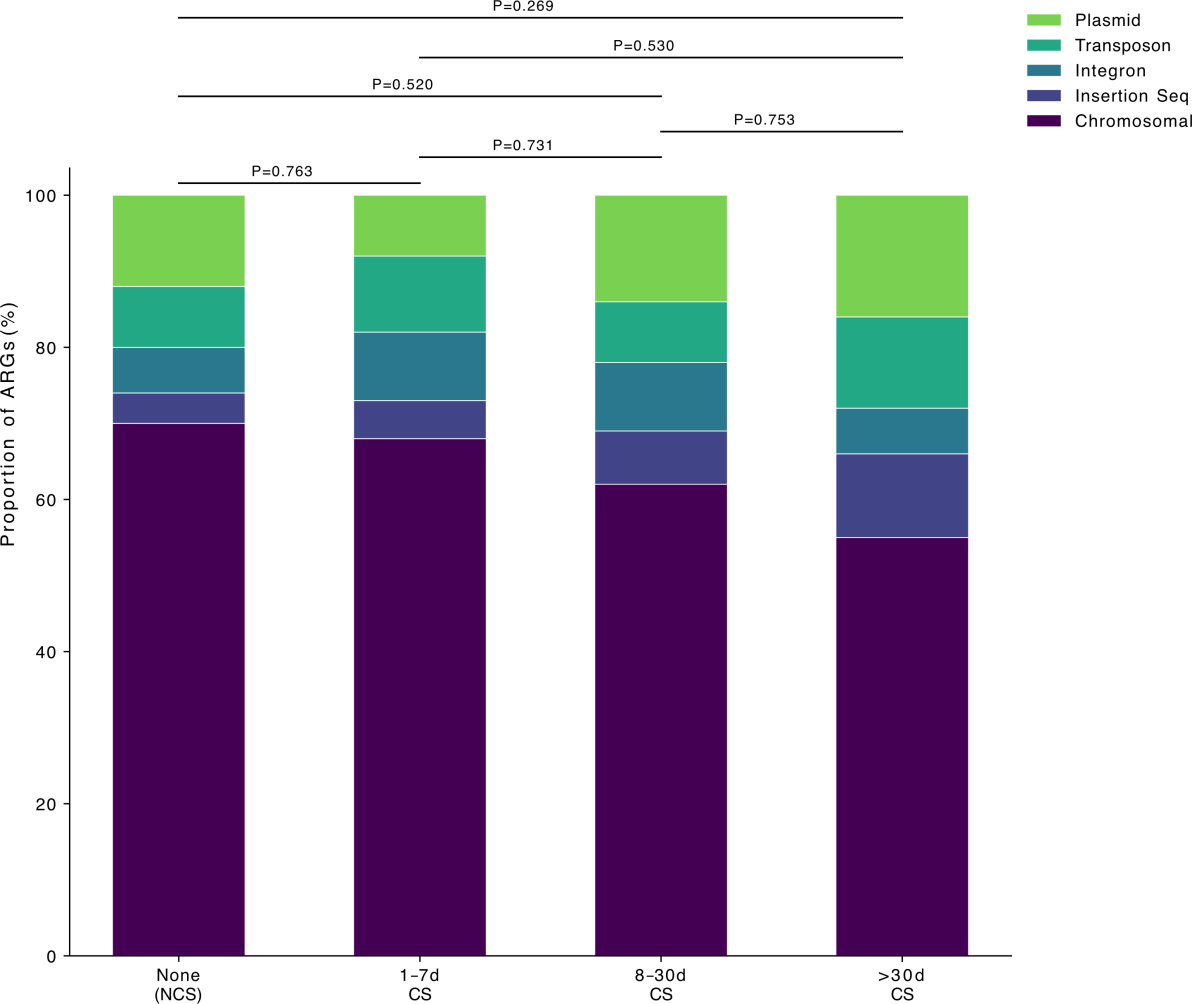
MGE-linked ARG ratios among corticosteroid duration categories. The proportions of ARGs associated with different mobile genetic element types—plasmid, transposon, integron, insertion sequence, and chromosomal—across four corticosteroid duration groups. No significant differences in MGE-associated ARG proportions were observed among any group pairs (all *P* > 0.05). A numerical trend indicating elevated MGE-associated ARG proportions was noted in the >30-day group; however, this did not achieve statistical significance. We used BWA-MEM alignment against mobileOG-db to determine whether ARGs and MGEs were present at the same time. ARG, antibiotic resistance gene; BWA, Burrows–Wheeler Aligner; CS, corticosteroid; MGE, mobile genetic element; NCS, non-corticosteroid.

There were no significant differences in the proportions of MGE-associated ARGs between the groups (none vs 1–7 days: *P* = 0.763; none vs 8–30 days: *P* = 0.731; none vs >30 days: *P* = 0.530; 1–7 days vs 8–30 days: *P* = 0.753; 1–7 days vs >30 days: *P* = 0.520; 8–30 days vs >30 days: *P* = 0.269) (Fig. 4). Although a minor numerical trend indicating elevated MGE-associated proportions was noted in the >30-day group, it did not achieve statistical significance. Chromosomal ARGs remained the most common type in all groups. These results suggest that the ARG buildup that happens with long-term use of corticosteroids is not due to a detectable change in mobile element carriage at the read level.

### Association of corticosteroid therapy with ARGs in lower respiratory tract

**Fig 5:**
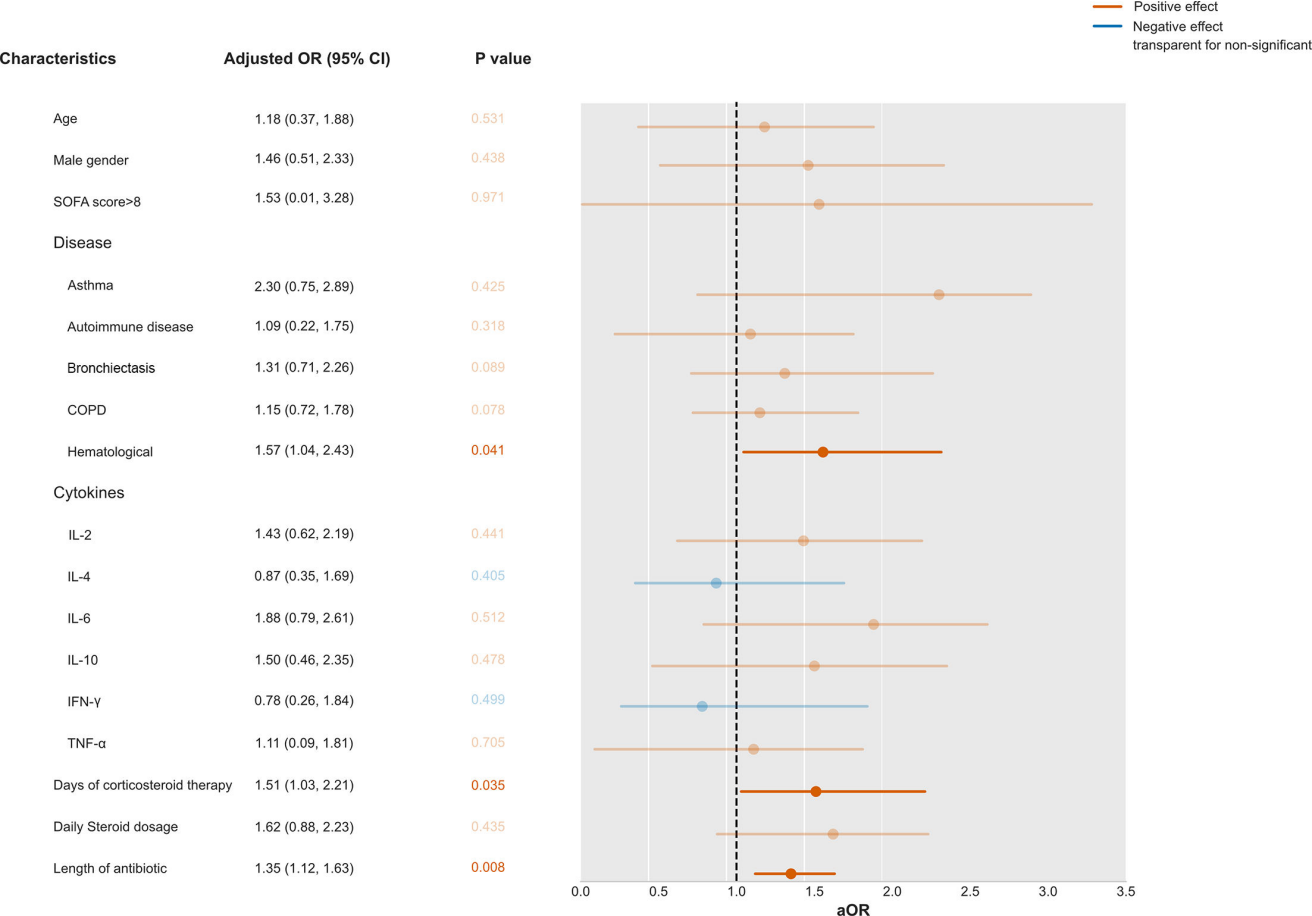
Multivariable logistic regression analysis of the association of corticosteroid therapy and ARG accumulation. Adjusted for age, gender, comorbidity, serum cytokines, and daily steroid dosage. Patients with longer duration of corticosteroid therapy had higher adjusted risks of ARG accumulation (adjusted odds ratio [aOR]: 1.51, 95% CI: 1.03–2.21, *P* = 0.035), and another independent risk factor was underlying hematological diseases (aOR: 1.57, 95% CI: 1.04–2.43, *P* = 0.041) and duration of antibiotic (aOR: 1.35, 95% CI: 1.12–1.63, *P* = 0.008). The association between antibiotic duration and ARG accumulation is likely bidirectional and should not be interpreted as directional causation. aOR, adjusted odds ratio; ARGs, antibiotic resistance genes; CI, confidence interval; COPD, chronic obstructive pulmonary disease; IL, interleukin; IFN-γ, interferon-γ; SOFA, sequential organ failure assessment; TNF, tumor necrosis factor.

Using multivariable logistic regression, patient features were analyzed for adjusted odds ratio of any ARG presentation (Fig. 5). Adjustments were made for age, gender, comorbidity, serum cytokines, and daily steroid dosage. Patients with longer duration of corticosteroid therapy had higher adjusted risk of ARG accumulation (adjusted odds ratio [aOR]: 1.51, 95% CI: 1.03–2.21, *P* = 0.035), and another independent risk factor was underlying hematological diseases (aOR: 1.57, 95% CI: 1.04–2.43, *P* = 0.041), as well as duration of antibiotic use (aOR: 1.35, 95% CI: 1.12–1.63, *P* = 0.008). The daily dosage of corticosteroids was not associated with ARG accumulation.

## DISCUSSION

In this study, we explored the ARG reservoir of critically ill patients with LRTIs. The metagenomic analysis revealed that prior corticosteroid (CS) use significantly alters ARGs, and patients with a history of corticosteroid therapy demonstrated a higher burden and a unique composition of ARGs, especially those with extended corticosteroid use over 30 days.

These findings correspond with growing evidence that corticosteroids—although therapeutically beneficial in mitigating inflammatory responses—can induce microbial dysbiosis and facilitate colonization by antibiotic-resistant pathogens (14, 15). Our research extends these findings to the ICU environment and presents direct metagenomic evidence suggesting that corticosteroid treatment may promote the accumulation of ARGs in the lower respiratory tract.

ARGs that significantly accumulated in the CS group were those specified to aminoglycosides, beta-lactams, and carbapenems. In a recent study (17) that reviewed the prescriptions of antibiotics for COPD patients in China, beta-lactam was the most frequently prescribed one. For immunocompromised patients in general, many guidelines and consensus statements recommend empirically extending the antibiotic spectrum in community-acquired pneumonia (CAP) (18). In summary, guideline recommendations and antibiotic application practices may have contributed to the accumulation of ARGs for specified antibiotics in patients with a history of routine corticosteroid therapy. From another perspective, the fluoroquinolones were the most frequently used antibiotics for CAP in China (19), which was supported by our observations and may account for the non-significant difference of fluoroquinolone ARGs between the two groups, as most enrolled patients may have received this type of antibiotic previously.

Microbial profiling indicated that corticosteroid-treated individuals possessed unique microbial communities enriched in taxa often linked to antimicrobial resistance and nosocomial infections. The detected increase in drug-resistant infections was positively associated with ARG load, further reinforcing a mechanistic connection between microbial dysbiosis and the pulmonary ARGs under corticosteroid influence. The accumulation of ARGs in CS-treated patients was possibly a result of enrichment of microbes with MGR potentials.

After adjustment for the demographical factors, underlying diseases, severity of organ dysfunctions (evaluated by SOFA score), and inflammatory cytokine profile, the duration of corticosteroid therapy was still associated with higher risk of ARG accumulation. Another independent risk factor identified in our logistic regression was the underlying hematological disease, which could be explained by a generally longer history of antibiotic exposure (20) and possibly more episodes of corticosteroid therapy in these patients (especially after hematopoietic stem cell transplantation [21]). Additionally, we identified a duration-dependent correlation between corticosteroid administration and ARG load, with markedly elevated ARG counts seen exclusively in individuals treated with corticosteroids for over 30 days. No connection was observed with daily corticosteroid dosage, indicating that the cumulative immunomodulatory effect over time, rather than short-term intensity, may more significantly influence resistance gene dynamics. The association between antibiotic duration and ARG accumulation identified in the regression warrants careful interpretation. Prolonged antibiotic exposure creates selective pressure favoring resistant organisms, but the reverse is equally plausible: patients harboring a higher ARG burden may require more prolonged antibiotic courses to achieve clinical resolution.

As a previous study has shown, corticosteroids can influence mucosal immunity (22, 23), which may further influence microbiota composition. Since MGE analysis showed no significant difference between groups in the length of corticosteroids in MEG proportion, horizontal gene transfer could not be the primary mechanism of ARG accumulation. The changes in species-level composition might be the main cause.

From a clinical standpoint, these findings necessitate a more nuanced evaluation of the previous corticosteroid use in patients with respiratory infections. Corticosteroids are essential for treating several inflammatory and immune-mediated disorders. However, their ability to worsen antimicrobial resistance by disrupting the lung microbiota must be acknowledged. In patients with LRTI and acute respiratory distress syndrome, corticosteroids have been proven effective (24). However, based on the results of the present study, extended corticosteroid treatment may elevate the likelihood of harboring multidrug-resistant pathogens, complicating infection care and perhaps influencing outcomes. Clinicians should at least consider the timely tapering of corticosteroids in patients with LRTIs (25).

This research possesses multiple limitations. The sample size, despite being prospectively gathered, is limited and may restrict generalizability. Furthermore, whereas metagenomic sequencing provides detailed insights into the ARGs, it cannot differentiate between gene expression and resistance phenotype. Second, our approach cannot distinguish between active infection and colonization. Future studies should combine metagenomic data with culture-based antimicrobial susceptibility testing and transcriptomic analysis to validate the functional significance of identified ARGs. Future research incorporating functional assays and longitudinal sampling is necessary to establish causal relationships between genotype and phenotype of antibiotic resistance in microbes and to elucidate temporal dynamics. Corticosteroid exposure was varied for indication, route, and formulation. Despite our efforts to stratify by duration, residual confounding remains possible. Our cross-sectional design measured ARG burden at ICU admission but was unable to evaluate temporal dynamics. We are uncertain if ARG accumulation continues after the cessation of corticosteroids or if tapering diminishes resistance gene burden. Longitudinal studies involving serial bronchoalveolar lavage (BAL) sampling during and after corticosteroid treatment are essential to ascertain: (i) the temporal dynamics of ARG accumulation during therapy, (ii) the persistence of ARGs following the cessation of corticosteroids, and (iii) the potential interventions that may expedite ARG clearance. Such data would assist in predicting individual patient risk for resistant infection. The route of corticosteroid administration was not analyzed separately because the subgroup sizes were small (oral *n* = 11; inhaled *n* = 8). Most patients (66.67%) were administered intravenous corticosteroids, suggesting that systemic immunosuppression may have facilitated the observed ARG associations. Subsequent research ought to investigate whether inhaled corticosteroids, which exert localized effects, influence lung ARG burden comparably.

In conclusion, our data present new evidence that in patients with LRTIs, extended corticosteroid use is associated with ARG accumulation and modifications in the microbial composition of the lower respiratory tract.

## Supplementary Material

Reviewer comments

## Data Availability

The raw sequencing data generated in this study have been deposited in the NCBI Sequence Read Archive (SRA) under accession number PRJNA1490573.

## SUPPLEMENTAL MATERIAL

OPEN PEER REVIEW reviewer-comments.pdf An accounting of the reviewer comments and feedback.

ASM does not own the copyrights to Supplemental Material that may be linked to, or accessed through, an article. The authors have granted ASM a non-exclusive, world-wide license to publish the Supplemental Material files. Please contact the corresponding author directly for reuse.
