# Supplementary material for: Impact of corticosteroids on lung antibiotic resistance genes in patients with lower respiratory tract infections
Source: Microbiol Spectr. 2026 Jul 15;14(8):e02080-25. doi: 10.1128/spectrum.02080-25 (PMC13435754; doi:10.1128/spectrum.02080-25)
Supplement: Supplementary file 1 [file reviewer-comments.pdf]

AMERICAN SOCIETY FOR MICROBIOLOGY | Microbiology Spectrum

Impact of Corticosteroid on Lung Antibiotic Resistance Genes in Patients with Lower Respiratory Tract Infection

Jiawei Shen, Yan Hu, Xiaoyun Zou, Xiujuan Zhao, Shu Li, Yanwen Jiang, and Fengxue Zhu

Corresponding Author(s): Fengxue Zhu, Peking University People's Hospital

Review Timeline:

Submission Date:	July 8, 2025
Editorial Decision:	January 18, 2026
Revision Received:	February 13, 2026
Editorial Decision:	April 22, 2026
Revision Received:	April 22, 2026
Accepted:	May 15, 2026

Editor: Jian Peng

Reviewer(s): Disclosure of reviewer identity is with reference to reviewer comments included in decision letter(s). The following individuals involved in review of your submission have agreed to reveal their identity: Michael Niederman (Reviewer #1)

Transaction Report:

(Note: With the exception of the correction of typographical or spelling errors that could be a source of ambiguity, letters and reports are not edited. The original formatting of letters and referee reports may not be reflected in this compilation.)

DOI: https://doi.org/10.1128/spectrum.02080-25

Re: Spectrum02080-25 (**Impact of Corticosteroid on Lung Antibiotic Resistance Genes in Patients with Lower Respiratory Tract Infection**)

Dear Dr. Fengxue Zhu:

Thank you for the privilege of reviewing your work. Below you will find my comments, instructions from the Spectrum editorial office, and the reviewer comments.

This study addresses a critical clinical gap by investigating corticosteroids' impact on lung antibiotic resistance genes (ARGs) in LRTI patients, with a rigorous prospective design and metagenomic sequencing. The findings-linking prolonged corticosteroid use to increased ARG diversity, enrichment of drug-resistant microbes, and duration-dependent ARG accumulation-provide valuable insights for antimicrobial stewardship. The multivariable regression adjusts for key confounders, strengthening causal inferences.
However, limitations include a modest sample size, lack of functional validation of ARG expression, and residual confounding from varied corticosteroid indications/routes. Despite these, the research offers timely evidence to inform clinical decision-making, urging cautious corticosteroid prescribing in LRTIs. It lays a foundation for future longitudinal studies to confirm ARG-phenotype relationships and optimize therapeutic strategies. There are still many problems in the paper that need to be corrected.

Specific Review Comments
1. The introduction briefly mentions corticosteroids' potential to alter microbiota but lacks a focused discussion of prior evidence linking immunosuppression to ARG dissemination, which would better frame the study's novelty.
2. Patient exclusion criteria do not specify whether prior antibiotic use (a key ARG driver) was accounted for; this confounding variable should be addressed in analysis or discussion.
3. The methods do not clarify how corticosteroid dosage was standardized across routes (inhaled, oral, intravenous), making it difficult to compare exposure intensity.
4. While metagenomic sequencing identifies ARGs, functional assays (e.g., antibiotic susceptibility testing) are absent to confirm if ARGs translate to phenotypic resistance.
5. The discussion does not explain why fluoroquinolone ARGs showed no group difference beyond general antibiotic use; stratifying by fluoroquinolone exposure could enhance clarity.
6. Baseline data on patients' prior respiratory infections or hospitalizations-factors influencing ARG burden-are not reported and should be replenish.
7. The study does not explore potential mechanisms (e.g., horizontal gene transfer frequency) underlying corticosteroid-induced ARG accumulation, which would deepen mechanistic insights.
8. Sample size justification is lacking; a power analysis should be added to validate the ability to detect group differences in ARG diversity.
9. The duration of corticosteroid use is categorized broadly (1-7 days, >30 days); finer stratification (e.g., 8-30 days) could refine the duration-response relationship.
10. Longitudinal data on ARG dynamics post-corticosteroid withdrawal are missing, limiting conclusions on reversibility of ARG accumulation.

Please return the manuscript within 60 days; if you cannot complete the modification within this time period, please contact me. If you do not wish to modify the manuscript and prefer to submit it to another journal, notify me immediately so that the manuscript may be formally withdrawn from consideration by Spectrum.

Revision Guidelines
To submit your modified manuscript, log into the submission site at https://spectrum.msubmit.net/cgi-bin/main.plex. Go to Author Tasks and click the appropriate manuscript title to begin. The information you entered when you first submitted the paper will be displayed; update this as necessary. Note the following requirements:

• Upload point-by-point responses to the issues raised by the reviewers in a file named "Response to Reviewers," NOT in your cover letter.
• Upload a compare copy of the manuscript (without figures) as a "Marked-Up Manuscript" file.
• Upload a clean .DOC/.DOCX version of the revised manuscript and remove the previous version.
• Each figure must be uploaded as a separate, editable, high-resolution file (TIFF or EPS preferred), and any multipanel figures must be assembled into one file.
• Any supplemental material intended for posting by ASM should be uploaded with their legends separate from the main manuscript. You can combine all supplemental material into one file (preferred) or split it into a maximum of 10 files with all associated legends included.

For complete guidelines on revision requirements, see our Submission and Review Process webpage. Submission of a paper that does not conform to guidelines may delay acceptance of your manuscript.

Data availability: ASM policy requires that data be available to the public upon online posting of the article, so please verify all links to sequence records, if present, and make sure that each number retrieves the full record of the data. If a new accession number is not linked or a link is broken, provide Spectrum production staff with the correct URL for the record. If the accession numbers for new data are not publicly accessible before the expected online posting of the article, publication may be delayed; please contact production staff (Spectrum@asmusa.org) immediately with the expected release date.

Publication Fees: For information on publication fees and which article types are subject to charges, visit ourwebsite. If your manuscript is accepted for publication and any fees apply, you will be contacted separately about payment during the production process; please follow the instructions in that e-mail. Arrangements for payment must be made before your article is published.

ASM Membership: Corresponding authors may join or renew ASM membership to obtain discounts on publication fees. Need to upgrade your membership level? Please contact Customer Service at Service@asmusa.org.

The ASM Journals program strives for constant improvement in our submission and publication process. Please tell us how we can improve your experience by taking this quick Author Survey.

Thank you for submitting your paper to Spectrum.

Sincerely,
Jian Peng
Editor
Microbiology Spectrum

Reviewer #1 (Comments for the Author):

this study looks at prospectively collected data from BAL in 91 ICU patients to evaluate the impact of corticosteroids (CS) on antibiotic resistance genes (ARG). The authors divided patients into those who received CS prior to admit and those who did not, and related the use of CS, and its duration to a higher frequency of ARGs. While the findings are potentially interesting, there are multiple issues with the data as reported, and unless these can be adequately addressed, the paper has limited value. Issues include: 1) it is unclear who these patients are and why they are in ICU, how sick they are, and if they were intubated. 2) it is not clear why they received CS for a prolonged period prior to admit. 3) most importantly, CS included inhaled, oral and intravenous use. There are no data about the frequency of each type of use, and no analysis about the impact of route of use on the effects observed. Is this an effect of both systemic and topical therapy , or only one type? 4) how was BAL done, how much fluid was instilled and how was it done in non-intubated patients? 5) how well matched are the CS and non-CS groups for severity of underlying illness that led to ICU admit?6) Are al ILRTIs an acute pneumonia, or are some infections in those with prior abnormal x rays? Did all patients receive antibiotic therapy ? 7) what is the impact of antibiotic use and hospitalization in the 30-90 days prior to the current admission, with regard to ARGs?8) which specific genes are included in the ARG panel? 9) the introduction talks about improving patient outcome based on the findings. I would omit this, since no outcome data are examined. 10) The methods section is out of place, and appears at the end of the paper

Response to reviewers

This study addresses a critical clinical gap by investigating corticosteroids' impact on lung antibiotic resistance genes (ARGs) in LRTI patients, with a rigorous prospective design and metagenomic sequencing. The findings-linking prolonged corticosteroid use to increased ARG diversity, enrichment of drug-resistant microbes, and duration-dependent ARG accumulation-provide valuable insights for antimicrobial stewardship. The multivariable regression adjusts for key confounders, strengthening causal inferences.
However, limitations include a modest sample size, lack of functional validation of ARG expression, and residual confounding from varied corticosteroid indications/routes. Despite these, the research offers timely evidence to inform clinical decision-making, urging cautious corticosteroid prescribing in LRTIs. It lays a foundation for future longitudinal studies to confirm ARG-phenotype relationships and optimize therapeutic strategies. There are still many problems in the paper that need to be corrected.

Specific Review Comments
1. The introduction briefly mentions corticosteroids' potential to alter microbiota but lacks a focused discussion of prior evidence linking immunosuppression to ARG dissemination, which would better frame the study's novelty.

Response: We agree the introduction could help the readers better understand the importance for performing this study. We have expanded the second paragraph, the new version includes more discussion on immunosuppression and ARG dynamics.

2. Patient exclusion criteria do not specify whether prior antibiotic use (a key ARG driver) was accounted for; this confounding variable should be addressed in analysis or discussion.

Response: Yes, we added the information of prior antibiotic usage in the study(in Table 1 and Figure 4). The usage percent of antibiotic were not significantly different in the two groups, but CS group had longer antibiotic therapies. In multivariable logistic resgression, length of antibiotic, together with days of corticosteroid therapy and hematological disease were independent predictors of any ARG accumulation.

3. The methods do not clarify how corticosteroid dosage was standardized across routes (inhaled, oral, intravenous), making it difficult to compare exposure intensity.

Response: Thank you for highlighting this methodological detail. The vast majority of patients received intravenous corticosteroids, which simplified dose comparison. Corticosteroid dosing was predominantly intravenous (38 of 57 patients, 66.67%. recorded in Table 1), with methylprednisolone being the most common agent. For patients receiving different formulations, we converted all doses to methylprednisolone equivalents using standard conversion ratios: prednisone (×1.25), dexamethasone (×0.15), hydrocortisone (×5), and inhaled budesonide (×40 for systemic equivalent). Daily dosage was calculated as the average methylprednisolone-equivalent dose over the treatment period (added to the method section).

4. While metagenomic sequencing identifies ARGs, functional assays (e.g., antibiotic susceptibility testing) are absent to confirm if ARGs translate to phenotypic resistance.

Response: We agree this represents a limitation. Metagenomic sequencing identifies genetic potential for resistance but cannot confirm whether genes are expressed or confer phenotypic resistance. We don't have the full access to the culture results. We have added this explicitly to the limitations.

5. The discussion does not explain why fluoroquinolone ARGs showed no group difference beyond general antibiotic use; stratifying by fluoroquinolone exposure could enhance clarity.

Response: We performed stratified analysis by fluoroquinolone exposure and found that fluoroquinolone usage lacks of group difference (revised Table 1). Actually both the CS and NCS patients had high percentage of fluroquinolone usage, which explained their were no fluroquinolone ARG difference. We have extended the discussion section on this topic.

6. Baseline data on patients' prior respiratory infections or hospitalizations-factors influencing ARG burden-are not reported and should be replenish.

Response: We have now included this important baseline information (Table 1), which provides context for patients' ARG burden.

7. The study does not explore potential mechanisms (e.g., horizontal gene transfer frequency) underlying corticosteroid-induced ARG accumulation, which would deepen mechanistic insights.

Response: We agree with you that mechanism analysis can provide more insights to the ARG accumulation phenomenon. We performed the mobile genetic elements (MGEs) analysis using sequencing reads. Result was depicted in new Figure 4. There were no obvious different of MGE proportion between NCS and different CS groups (1-7 days to over 30 days). all pairwise comparisons P>0.05, see new Figure 4). While a numerical trend toward higher MGE proportions was observed in the >30d group, this did not reach statistical significance. Chromosomal ARGs remained dominant across all groups. These findings suggest the ARG accumulation associated with prolonged corticosteroid use is not explained by a detectable shift toward mobile element carriage at the read level. We have added this analysis to the Methods, Results, and Discussion.

8. Sample size justification is lacking; a power analysis should be added to validate the ability to detect group differences in ARG diversity.

Response: We acknowledge that a priori sample size calculations were not performed, this study was an exploratory one of a question that had never been studied before. We have now calculated the post-hoc power based on the effect sizes we observed. Based on ARG counts between the CS and NCS groups (n=57 vs n=34), the effect size that evaluated with Cohen's d was 0.54, which is a medium effect. The achieved power was 70% at α=0.05. This is less than

the usual 80% threshold, but it's good enough for an exploratory study. The significant result (Mann-Whitney U test, P=0.014), despite limited power, indicates that the association is genuine rather than a false positive.

9. The duration of corticosteroid use is categorized broadly (1-7 days, >30 days); finer stratification (e.g., 8-30 days) could refine the duration-response relationship.

Response: We appreciate the suggestion. The manuscript already includes four-category stratification (None, 1-7 days, 8-30 days, >30days), as shown in Figure 2c. No additional manuscript revision was made.

10. Longitudinal data on ARG dynamics post-corticosteroid withdrawal are missing, limiting conclusions on reversibility of ARG accumulation.

Response: We agree longitudinal sampling would provide valuable information about ARG persistence and reversibility. This represents a limitation of our cross-sectional design and was recoreded in the limitation section.

Reviewer 1:

1.it is unclear who these patients are and why they are in ICU, how sick they are, and if they were intubated.

Response: Thank you for reviewing our manuscript. As the criteria for the study described, these were ICU patients with respiratory failure requiring close monitoring or ventilatory support. All met both clinical and radiological criteria for LRTI. Illness severity was similar between groups by SOFA score (CS vs. NCS: 5[3-9] vs. 4[3-7], P=0.396). Intubation rates were higher in the CS group, reflecting the higher proportion of immunocompromised patients with hematological diseases and autoimmune conditions. We added intubation status to Table 1.

2. it is not clear why they received CS for a prolonged period prior to admit.

Response: Prolonged corticosteroid use was driven by chronic conditions requiring long-term therapy. The main indications were COPD (n=22, 38.6%), autoimmune diseases (n=13, 22.8%), and hematological diseases including post-transplant graft-versus-host disease (n=12, 21.1%). These are already in Table 1. We added a clarifying sentence to Results.

3. most importantly, CS included inhaled, oral and intravenous use. There are no data about the frequency of each type of use, and no analysis about the impact of route of use on the effects observed. Is this an effect of both systemic and topical therapy , or only one type?

Response: Route distribution was: IV 38 (66.67%), oral 11 (19.29%), inhaled 8 (14.04%). We added this to Table 1. Route-stratified analysis would require larger subgroups — with only 11 oral and 8 inhaled patients, separate analysis risks misleading results. The predominance of IV use indicates systemic immunosuppression was the main exposure. All doses were converted to methylprednisolone equivalents for regression analysis. We added this limitation to Discussion.

4. how was BAL done, how much fluid was instilled and how was it done in non-intubated patients?

Response: We agree that the standarized protocol will ensure the reproducibility of the research.We expanded the BAL protocol description. The same standardized protocol was used at both centers.

5. how well matched are the CS and non-CS groups for severity of underlying illness that led to ICU admit?

Response: Severity was similar. SOFA scores did not differ (CS: 5[3-9] vs. NCS: 4[3-7], P=0.396), and SOFA was included in the regression model. The CS group had lower inflammatory markers (CRP, IL-6, TNF-α), and lower lymphocyte counts — findings consistent with corticosteroid immunosuppression rather than different infection severity. These immune differences were also adjusted in regression.

6. Are all LRTIs an acute pneumonia, or are some infections in those with prior abnormal x rays? Did all patients receive antibiotic therapy?

Response: The cohort was mixed. Most had acute pneumonia, but some had prior lung pathology including COPD-related changes and post-transplant pulmonary complications with pre-existing radiological abnormalities. Prior pathology was distributed similarly between groups

(Table 1 comorbidities). All 91 patients received antibiotics during ICU admission per standard LRTI protocols. We added the relevent contents to the Methods section.

7. what is the impact of antibiotic use and hospitalization in the 30-90 days prior to the current admission, with regard to ARGs?

Response: We collected data on prior antibiotic exposure within 30 days before ICU admission (data out of that range was not accessible) and hospitalization within prior year before ICU admission. Prior antibiotic use was similar between groups (CS: 78.9% vs. NCS: 73.5%, P=0.55), as was prior hospitalization (CS: 66.67% vs. NCS: 55.88%, P=0.301). We added antibiotic treatment duration (length of antibiotic therapy) to the multivariable logistic regression model. Antibiotic duration was independently associated with ARG accumulation (aOR: 1.35, 95% CI: 1.12-1.63, P=0.008), confirming this is an important confounder.

However, corticosteroid duration remained independently significant after adjusting for antibiotic exposure (aOR: 1.51, 95% CI: 1.03-2.21, P=0.035), as did hematological disease (aOR: 1.57, 95% CI: 1.04-2.43, P=0.041). These results demonstrate that the corticosteroid-ARG association persists even after accounting for antibiotic use.

Given the sample size constraints and number of existing covariates in the multivariable model, we did not add prior hospitalization and antibiotic usage (which were also not significantly different between the two groups) into the logistic regression to prevent overfitting of the model.

8. which specific genes are included in the ARG panel?

Response: ARG identification used RGI v6.0.3 against CARD v4.0.0 (https://card.mcmaster.ca). CARD is a publicly accessible, peer-reviewed database of resistance determinants. In our cohort, 86 ARGs across 9 resistance classes were identified. Their distribution is shown in Figure 2e. Referencing CARD directly is more informative than listing individual genes. The database provides detailed mechanistic information for each entry.

9. the introduction talks about improving patient outcome based on the findings. I would omit this, since no outcome data are examined.

Response: We agree with you that outcome data were not present, so we removed the outcome language from the Abstract and Introduction.

10. The methods section is out of place, and appears at the end of the paper

Response: Thank you and we have reodered the sections.

Re: Spectrum02080-25R1 (**Impact of Corticosteroid on Lung Antibiotic Resistance Genes in Patients with Lower Respiratory Tract Infection**)

Dear Dr. Fengxue Zhu:

Thank you for the privilege of reviewing your work. Below you will find my comments, instructions from the Spectrum editorial office, and the reviewer comments.

Please return the manuscript within 60 days; if you cannot complete the modification within this time period, please contact me. If you do not wish to modify the manuscript and prefer to submit it to another journal, notify me immediately so that the manuscript may be formally withdrawn from consideration by Spectrum.

Revision Guidelines
To submit your modified manuscript, log into the submission site at https://spectrum.msubmit.net/cgi-bin/main.plex. Go to Author Tasks and click the appropriate manuscript title to begin. The information you entered when you first submitted the paper will be displayed; update this as necessary. Note the following requirements:

• Upload point-by-point responses to the issues raised by the reviewers in a file named "Response to Reviewers," NOT in your cover letter.
• Upload a compare copy of the manuscript (without figures) as a "Marked-Up Manuscript" file.
• Upload a clean .DOC/.DOCX version of the revised manuscript and remove the previous version.
• Each figure must be uploaded as a separate, editable, high-resolution file (TIFF or EPS preferred), and any multipanel figures must be assembled into one file.
• Any supplemental material intended for posting by ASM should be uploaded with their legends separate from the main manuscript. You can combine all supplemental material into one file (preferred) or split it into a maximum of 10 files with all associated legends included.

For complete guidelines on revision requirements, see our Submission and Review Process webpage. Submission of a paper that does not conform to guidelines may delay acceptance of your manuscript.

Data availability: ASM policy requires that data be available to the public upon online posting of the article, so please verify all links to sequence records, if present, and make sure that each number retrieves the full record of the data. If a new accession number is not linked or a link is broken, provide Spectrum production staff with the correct URL for the record. If the accession numbers for new data are not publicly accessible before the expected online posting of the article, publication may be delayed; please contact production staff (Spectrum@asmusa.org) immediately with the expected release date.

Publication Fees: For information on publication fees and which article types are subject to charges, visit our website. If your manuscript is accepted for publication and any fees apply, you will be contacted separately about payment during the production process; please follow the instructions in that e-mail. Arrangements for payment must be made before your article is published.

ASM Membership: Corresponding authors may join or renew ASM membership to obtain discounts on publication fees. Need to upgrade your membership level? Please contact Customer Service at Service@asmusa.org.

The ASM Journals program strives for constant improvement in our submission and publication process. Please tell us how we can improve your experience by taking this quick Author Survey.

Thank you for submitting your paper to Spectrum.

Sincerely,
Jian Peng
Editor
Microbiology Spectrum

Reviewer #1 (Comments for the Author):

Thank you for your revisions. You have very nicely answered my questions and concerns. This is a well written paper describing the relationship of pre admission corticosteroid use to the development of antibiotic resistance in ICU patients with LRTI , all treated with antibiotics. You have nicely shown a relationship between corticosteroid use prior to admission and antibiotic resistance genes, showing a relationship of resistance genes to duration of steroid use, but not dose used. Most patients got

systemic corticosteroid therapy, but the data set included a small number getting inhaled corticosteroids.
I have 2 additional suggestions for modification
1) On page 11, you discuss how the data in the study could be used to optimize steroid tapering and to predict antibiotic resistance. I would omit mention to steroid tapering, since patients needed steroids due to comorbid illness, and tapering at a time of acute illness, would not be a good management strategy.
2) In Figure 5 and in table 1, and in the text , you report that corticosteroid treated patients with a longer duration of antibiotic therapy had a greater risk of accumulating ARGS, and imply that prolonged antibiotic use could be a confounding cause of resistance. I would reword, since prolonged duration of therapy could be EITHER a cause or effect of antibiotic resistance.

Response to reviewers

Reviewer #1 (Comments for the Author):

Thank you for your revisions. You have very nicely answered my questions and concerns. This is a well written paper describing the relationship of pre admission corticosteroid use to the development of antibiotic resistance in ICU patients with LRTI , all treated with antibiotics. You have nicely shown a relationship between corticosteroid use prior to admission and antibiotic resistance genes, showing a relationship of resistance genes to duration of steroid use, but not dose used. Most patients got systemic corticosteroid therapy, but the data set included a small number getting inhaled corticosteroids.

I have 2 additional suggestions for modification

***1)** On page 11, you discuss how the data in the study could be used to optimize steroid tapering and to predict antibiotic resistance. I would omit mention to steroid tapering, since patients needed steroids due to comorbid illness, and tapering at a time of acute illness, would not be a good management strategy.*

Response: We agree with you that the tapering of steroid was mainly guided by comorbid illness severity. We have removed the contents of “guiding steroid tapering” in discussion.

2) In Figure 5 and in table 1, and in the text , you report that corticosteroid treated patients with a longer duration of antibiotic therapy had a greater risk of accumulating ARGS, and imply that prolonged antibiotic use could be a confounding cause of resistance. I would reword, since prolonged duration of therapy could be EITHER a cause or effect of antibiotic resistance.

Response: We thank the reviewer for this important point. We agree that the association between antibiotic duration and ARG accumulation is observational and cannot establish directionality. Prolonged antibiotic exposure may select for resistant organisms, but a higher ARG burden may itself necessitate longer treatment courses. The original framing implied a causal direction that the data do not support.

We have revised the relevant text in two locations:

> **Figure 5 legend:** A sentence was appended after the aOR for antibiotic duration: “The association between antibiotic duration and ARG accumulation is likely bidirectional and should not be interpreted as directional causation.”
>
> **Discussion** — after "...may more significantly influence resistance gene dynamics", we added: “The association between antibiotic duration and ARG accumulation identified in the regression warrants careful interpretation. Prolonged antibiotic exposure creates selective pressure favoring resistant organisms, but the reverse is equally plausible: patients harboring a higher ARG burden may require more prolonged antibiotic courses to achieve clinical resolution.”

Re: Spectrum02080-25R2 (**Impact of Corticosteroid on Lung Antibiotic Resistance Genes in Patients with Lower Respiratory Tract Infection**)

Dear Dr. Fengxue Zhu:

Your manuscript has been accepted, and I am forwarding it to the ASM production staff for publication. Your paper will first be checked to make sure all elements meet the technical requirements. ASM staff will contact you if anything needs to be revised before copyediting and production can begin. Otherwise, you will be notified when your proofs are ready to be viewed.

Data Availability: ASM policy requires that data be available to the public upon online posting of the article, so please verify all links to sequence records, if present, and make sure that each number retrieves the full record of the data. If a new accession number is not linked or a link is broken, provide production staff with the correct URL for the record. If the accession numbers for new data are not publicly accessible before the expected online posting of the article, publication may be delayed; please contact ASM production staff immediately with the expected release date.

Publication Fees: For information on publication fees and which article types have charges, please visit ourwebsite. We have partnered with Copyright Clearance Center (CCC) to collect author charges. If fees apply to your paper, you will receive a message from no-reply@copyright.com with further instructions. For questions related to paying charges through RightsLink, please contact CCC at ASM_Support@copyright.com or toll free at +1-877-622-5543. CCC makes every attempt to respond to all emails within 24 hours.

ASM Membership: Corresponding authors may join or renew ASM membership to obtain discounts on publication fees. Need to upgrade your membership level? Please contact Customer Service at Service@asmusa.org.

Embargo Policy: A press release may be issued as soon as the manuscript is posted on theSpectrum Latest Articles webpage. The corresponding author will receive an email with the subject line "ASM Journals Author Services Notification" when the article is available online.

The ASM Journals program strives for constant improvement in our submission and publication process. Please tell us how we can improve your experience by taking this quick Author Survey.

Thank you for submitting your paper to Spectrum.

Sincerely,
Jian Peng
Editor
Microbiology Spectrum

Reviewer #1 (Comments for the Author):

Thanks for your changes. I have no other suggestions and appreciate your efforts to clarify your message
